# The potential harms of primary human papillomavirus screening in over-screened women: a microsimulation study

**DOI:** 10.1007/s10552-016-0732-7

**Published:** 2016-03-12

**Authors:** Steffie K. Naber, Inge M. C. M. de Kok, Suzette M. Matthijsse, Marjolein van Ballegooijen

**Affiliations:** Department of Public Health, Erasmus Medical Center, P.O. Box 2040, 3000 CA Rotterdam, The Netherlands

**Keywords:** Uterine cervical neoplasms, Computer simulation, Early detection of cancer, Papanicolaou test, Human papillomavirus DNA tests

## Abstract

**Background:**

It is well acknowledged that HPV testing should not be performed at young age and at short intervals. Cytological screening practices have shown that over-screening, i.e., from a younger age and at shorter intervals than recommended, is hard to avoid. We quantified the consequences of a switch to primary HPV screening for over-screened women, taking into account its higher sensitivity but lower specificity than cytology.

**Methods:**

The health effects of using the HPV test instead of cytology as the primary screening method were determined with the MISCAN-Cervix model. We varied the age women start screening and the interval between screens. In the sensitivity analyses, we varied the background risk of cervical cancer, the HPV prevalence, the discount rate, the triage strategy after cytology, and the test characteristics of both cytology and the HPV test.

**Results:**

For women screened 5 yearly from age 30, 32 extra deaths per 100,000 simulated women were prevented when switching from primary cytology to primary HPV testing. For annual screening from age 20, such a switch resulted in 6 extra deaths prevented. It was associated with 9,044 more positive primary screens in the former scenario versus 76,480 in the latter. Under all conditions, for women screened annually, switching to HPV screening resulted in a net loss of quality-adjusted life years.

**Conclusion:**

For over-screened women, the harms associated with a lower test specificity outweigh the life years gained when switching from primary cytology to primary HPV testing. The extent of over-screening should be considered when deciding on inclusion of primary HPV screening in cervical cancer screening guidelines.

**Electronic supplementary material:**

The online version of this article (doi:10.1007/s10552-016-0732-7) contains supplementary material, which is available to authorized users.

## Introduction

In several Western countries, cytological screening has considerably reduced the cervical cancer incidence and mortality over the past four decades [1]. Nevertheless, even in countries with a nationwide screening program, women still die from cervical cancer. Although most deaths occur after age 30 and in women who did not adequately participate in screening, some deaths occur at young age and in women who recently received a negative test result (which suggests it was false negative) [2–4]. Therefore, clinicians may tend to screen more frequently than recommended [5].

Ever since infection with the human papillomavirus (HPV) was found to be a necessary condition for developing cervical cancer [6, 7], testing for the presence of high-risk HPV types (i.e., carcinogenic types) has received much attention. A summary of meta-analyses estimated that the HPV test has a 23 % (95 % CI 13–33 %) higher sensitivity, but a 6 % (95 % CI 4–8 %) lower specificity than cytology for detecting high-grade lesions and cervical cancer [8]. Cost-effectiveness analyses based on these findings have shown that in well-controlled screening situations primary HPV screening is likely to be more effective, as well as more cost-effective than primary cytology [9, 10]. Therefore, many countries are considering a switch from primary cytology to primary HPV screening. In the USA, co-testing (i.e., cytology combined with HPV testing) is already recommended, and Australia and the Netherlands are preparing a switch from primary cytology to primary HPV screening [11–13].

For primary cytology, it is known that over-screening, here defined as screening from a younger age or at shorter intervals than recommended, is neither required to detect progressive lesions in an early phase nor desired as it detects many regressive lesions. Unavoidably, it also involves more false-positive test results, adding to the psychological stress women may experience from having a positive test and being referred for colposcopy [14]. In addition, the costs of over-screening are substantial, amounting to approximately 0.5–1 billion USD per year for the US healthcare system, while yielding little or no health gains [15].

Because of its lower specificity to detect clinically relevant lesions, avoiding over-screening is even more essential for HPV screening than for cytology screening. The vast majority of HPV infections clear spontaneously, especially at young age [16]. Detecting these infections leads to unnecessary triage situations or referrals to colposcopy. For over-screened women, switching to HPV (co-)testing may therefore do more harm than good.

Guidelines driven by rational decision making tend to restrict cytology screening—and HPV screening even more so. The US guidelines currently recommend cervical screening in women aged 21–65 years with an interval of 3 or 5 years (dependent on both age and test) [17]. In European guidelines, primary HPV screening is recommended for women aged ≥35 and discouraged for those below the age of 30 [18]. In the Netherlands, primary HPV screening will be offered from age 30 to 65 every 5–10 years, and in Australia from age 25 to 69 every 5 years [11, 13]. Unfortunately, also for HPV screening, having well-considered screening policy recommendations will not guarantee that women are screened accordingly.

A recent US study showed that over 68 % of physicians would recommend another cytological test in 1 or 2 years where the guidelines recommend a 3-year interval [19]. After a negative co-test, 67–94 % of clinicians recommended a shorter screening interval than suggested by US guidelines [20]. Several European countries also have reported considerable over-screening [21]. In summary, large proportions of women are being over-screened with cytology, and this is likely to continue when HPV screening is implemented.

Notwithstanding these facts, HPV testing is, for good reasons, increasingly often included in primary screening recommendations. However, despite its lower specificity, we are unaware of intensified efforts to minimize the level of over-screening. In this study, we aim to quantify the harms and benefits of introducing primary HPV screening for women with diverse screening behaviors, age of first screen ranging from 20 to 30 years, and screening interval from 1 to 5 years. These scenarios cover both recommended schedules and observed levels of over-screening. The results of this study show the effects of introducing HPV screening for over-screened women, as well as for those who adhere to guidelines. Although the model was based on Dutch data, the resulting outcomes are important for all over-screened women, regardless of where they live. Since it seems too early to draw conclusions on the effect of switching to HPV screening in over-screened women who have been vaccinated, this analysis only considers unvaccinated cohorts.

## Methods

Health effects of different screening scenarios were estimated using the MISCAN-Cervix model, which is described in more detail in the model profile (see Supplement) [22].

### MISCAN-Cervix model

MISCAN-Cervix is a microsimulation model in which a large study population with individual life histories is generated. In all of the analyses presented here, we simulated a 20-year-old cohort of 100 million women with life expectancy as observed in the Netherlands [23], which was not affected by HPV vaccination (neither directly nor through herd immunity). A fraction of these women will acquire HPV infections and/or develop cervical intraepithelial neoplasia (CIN) lesions. If these precursors progress to cervical cancer, the result may be death. Screening can detect the disease, which can then be treated at an earlier stage. As a result, cervical cancer death may be prevented or postponed.

In the model, the disease development is in seven sequential stages: high-risk HPV infection, three preinvasive stages (CIN grades I, II, and III), and three invasive stages (International Federation of Gynecology and Obstetrics (FIGO) stages IA, IB, and II or worse). While preinvasive and FIGO IA stages can be diagnosed only by screening, because at these stages the women are assumed to be symptom-free, FIGO IB or worse can also be clinically diagnosed. Because precursors are usually not progressive [24], over 90 % of modeled HPV infections clear without ever resulting in neoplasia and most preinvasive lesions regress spontaneously. In the hypothetical situation without competing other-cause mortality, undetected preclinical invasive neoplasia will always progress to clinical cancer. CIN grades I and II can develop in the absence of a high-risk HPV infection; in that case, the lesion will always regress. CIN grade III or worse can only develop if a high-risk HPV infection is present.

### Triage strategies

For primary HPV screening and primary cytology, we used a cost-effective triage strategy, as published previously [9]. Primary cytological test results classified as atypical squamous cells of undetermined significance (ASCUS) or low-grade squamous intraepithelial lesion are immediately followed by an HPV test using the same material. A positive primary HPV test is immediately followed by cytology using the same material. If no cytological abnormalities are found, another cytological test is performed after 6 months.

Although the latter strategy will also be implemented in the Dutch screening program in 2017, triage strategies were not selected based on guidelines or current practice. Instead, we decided to select strategies based on cost-effectiveness, such that inefficiencies in triage strategies would not dilute or exaggerate the effect of switching to HPV screening. The triage practices of over-screened women are unknown and might be very heterogeneous. It seems unlikely that women, who do not follow primary screening guidelines, do follow the exact triage recommendations. We therefore chose to simulate a relatively simple triage strategy for both primary tests and to focus on the number of positive primary tests (i.e., those that require follow-up) instead of on the number of triage tests.

### Screening scenarios

We simulated 12 cohorts with different screening behaviors, varying the age at which women start screening (20, 25, or 30 years) and the frequency with which they get tested (every 1, 2, 3, or 5 years). In all scenarios, screening was assumed to end at or before the age of 65 [17, 25]. The resulting outcomes are only relevant for women having the screening behavior as modeled and should not be translated to an entire population.

### Assumptions for screening and treatment

Table 1 presents the base case assumptions for screening. We assumed the sensitivity of cytology (that is, the probability that the result is at least ASCUS) to be 40 % for true stage CIN grade I, 50 % for CIN grade II, and 75 % for CIN grade III or cancer [26]. In the model calibration, the sensitivity of testing for at least high-grade squamous intraepithelial lesion (HSIL), the cytological cutoff for referral to colposcopy, and therefore for detection, was estimated to be 4 % for CIN grade I, 18 % for CIN grade II, 56 % for CIN grade III, and 60 % for cervical cancer. Furthermore, the specificity of cytology was estimated to be 97.6 % based on Dutch data [27]. Based on the observed difference in CIN grade III or cancer detection rates between cytology and the HPV test, we assumed the sensitivity of the HPV test to be 94 % for a high-risk HPV infection [28]. As we assumed that cervical cancer can only develop if an HPV infection is present, the sensitivity for cervical cancer is also 94 %. The overall sensitivity for CIN lesions is lower and depends on the age-specific prevalence of HPV infections in CIN lesions. In the model, the specificity for detecting high-risk HPV infections was assumed to be 100 %. A probable (but unknown) lack of specificity was accounted for by the inclusion of fast-clearing infections, in concordance with HPV clearing studies [29, 30].

**Table 1:** Base case model inputs and variations in the sensitivity analyses

Parameter	Base case value	Alternative value(s)
Background risk of cervical cancer mortality	5 per 100,000 life years	10 per 100,000 life years
HPV prevalence in women without CIN grade II or worse^a^	Low	High^b^
*Sensitivity of cytology*		
Probability of at least ASCUS (at least triage) for:		
CIN grade I	40 % [26]	32 %
CIN grade II	50 % [26]	40 %
CIN grade III or worse	75 % [26]	60 %
Probability of at least HSIL (referral for colposcopy) for:		
CIN grade I	4 %^c^	3 %
CIN grade II	18 %^c^	14 %
CIN grade III	56 %^c^	45 %
Cervical cancer	60 %^c^	48 %
Specificity of cytology (CIN grade I or worse)	97.6 %^c^	95.2 %
Sensitivity of HPV test^d^	94 % [28]	85 % [8], 100 % [8]
Specificity of HPV test	100 %^e^	Not varied as such^f^
Discounting	3 % [36]	0 %, 5 %

*HPV* human papillomavirus, *CIN* cervical intraepithelial neoplasia, *ASCUS* atypical squamous cells of undetermined significance, *HSIL* high-grade squamous intraepithelial lesion

^a^Depends on age, age dependency was not varied

^b^The number of false-positive referrals to colposcopy and CIN grade I lesions was doubled

^c^Value was determined in model calibration

^d^Probability to detect an HPV infection, regardless of whether a CIN lesion or cancer is present

^e^A possible lack of specificity was modeled by including fast-clearing HPV infections

^f^As a lower specificity of the HPV test corresponds with a higher prevalence of harmless HPV infections in the model, this parameter was not varied

Detection and management of preinvasive lesions, including treatment if necessary, were assumed to lead to a 100 % cure rate. However, new HPV infections and recurring CIN lesions after CIN treatment cannot be excluded. For invasive cancer, we determined age-specific and stage-specific survival probabilities based on data from the Netherlands Cancer Registry [31]. Since cancers detected by screening are usually at a less advanced stage than clinically diagnosed ones, women have a higher chance to survive them. If an invasive cancer is screen-detected, the probability to die from cervical cancer is reduced by 89.4, 50, and 20 % when detected in FIGO stages IA, IB, and II or worse, respectively.

Table 2 presents the utility losses assumed in the base case scenario. A small (psychological) loss in quality of life is assumed for attending a screen (including waiting for the result) and for being in triage (including attending follow-up screenings). Larger losses in quality of life are assumed for being diagnosed and treated for CIN or cancer and for having a terminal stage of cervical cancer. We based the utility losses on nationally and internationally published data [32–35].

**Table 2:** Model inputs regarding the utility loss due to screening, treatment, and terminal care

	Disutility	Duration	Quality-adjusted time lost
Screening [34]			
Primary screening	0.005	2 weeks	2 hours
Being in triage	0.005	0.5 year^a^	22 hours
False-positive referral	0.005	0.5 year	22 hours
Treatment of preinvasive lesions [33 **]**			
CIN grade I	0.03	0.5 year	6 days
CIN grade II or III	0.07	1 year	26 days
Cancer treatment [32, 33] and terminal care [35 **]**			
FIGO stage I	0.062	5 years	4 months
FIGO stage II+	0.280	5 years	17 months
Terminal care	0.740	1 year	9 months

*CIN* cervical intraepithelial neoplasia, *FIGO* International Federation of Gynecology and Obstetrics

^a^Time between primary and triage test is 6 month

### Base case analysis

For every scenario, we first estimated health effects of both primary cytology and primary HPV testing as compared to the situation without screening. Then, differences in health effects between these two interventions were explored. A first indication of the harm–benefit balance of introducing primary HPV testing is given by the number of additional positive primary screens (i.e., at least ASCUS for cytology screening and HPV positive for HPV screening) that is required to prevent one additional cervical cancer death. As women with a positive primary screen require follow-up in terms of triage or colposcopy, we refer to this outcome measure as “Number Needed to Follow-up” or NNF.

Comparing the life years lost to cervical cancer between the two interventions yields the number of life years gained by switching to the more sensitive primary HPV testing. Similarly, the difference in total disutility due to screening and treatment caused by these interventions can be computed. As the number of quality-adjusted life years (QALYs) gained combines these positive and negative effects of screening, this outcome measure was used to compare the total health effects of primary HPV screening with those of primary cytology. Health effects were discounted to the year in which all women are 20 years old, using an annual rate of 3 % [36].

### Sensitivity analyses

Some model parameters may have a non-negligible level of uncertainty, while others differ among countries or geographical regions. In one-way sensitivity analyses, we varied these types of parameters, covering for high-income countries, if they would influence the difference in health effects between primary HPV screening and primary cytology (Table 1).

Among Dutch women, the assumed background risk of dying from cervical cancer is relatively low (5 deaths per 100,000 life years). We have doubled this risk to determine the effects for countries with a higher risk.

To observe the effect of a higher prevalence of harmless HPV infections, we have doubled the number of referrals that did not result in the detection of a clinically relevant lesion (i.e., CIN grade II or worse). Detecting more harmless HPV infections implicitly corresponds with a lower clinically relevant specificity of HPV testing.

Presumably, the high level of quality assurance in the Netherlands contributes to a relatively high quality of cytology compared to less controlled situations. To explore the impact of switching to HPV testing for settings with a lower quality of cytology, the sensitivity of cytology in both primary and triage testing was reduced by 20 % in one of the sensitivity analyses. In another sensitivity analysis, the lack of specificity of cytology in both primary and triage testing was doubled from 2.4 to 4.8 %.

Some uncertainty exists about the sensitivity of the HPV test, which may also vary between tests and situations. A summary of meta-analyses found that the relative sensitivity of the HPV test as compared to cytology is 1.23 (95 % CI 1.13–1.33). Based on this confidence interval, the sensitivity of the HPV test was assumed to be 85 % in one of the sensitivity analyses and 100 % in another [8]. As these are assumed probabilities to detect an HPV infection, and women with a CIN lesion are not necessarily HPV infected, the sensitivity for CIN lesions is still lower than 100 % in the latter scenario.

In another sensitivity analysis, the triage strategy after a positive cytological test was adjusted to reflect current Dutch screening guidelines. According to these guidelines, women with HSIL are directly referred for colposcopy and women with ASCUS or low-grade intraepithelial lesion (LSIL) are invited for cytology and HPV triage after 6 months. Women testing HSIL or ASCUS/LSIL and HPV positive at this point in time will be referred for colposcopy, and women testing either ASCUS/LSIL or HPV positive will be invited for another cytological test at 18 months.

Lastly, as reported discount rates vary from 0 to 5 %, we also present the health effects when using an annual discount rate of 0 % and of 5 %.

## Results

### Base case analysis

For the 12 different screening scenarios considered, Table 3 shows the impact of replacing primary cytology with primary HPV screening. The numbers are based on the undiscounted results of primary cytology and primary HPV screening compared to the situation without screening, as displayed in Supplementary Tables 1 and 2, respectively. Although in practice, it is very unlikely that the start age is well controlled, while the screening interval is not, we first discuss the effects of switching to HPV testing in women who start screening at age 30 and have repeated testing at intervals that are either recommended or shorter than recommended. Then, we discuss the effects of switching for women who are not only screened more frequent than recommended, but also from a younger age.

**Table 3:** The impact of replacing primary cytology with primary HPV screening for 12 different screening scenarios

Screening interval	Start age	# Primary screens^a^	# Positive primary screens	# Referrals to colposcopy	# False-positive referrals (no CIN detected)	# CIN grade I	# CIN grade II	# CIN grade III	# Cervical cancer cases	# Cervical cancer deaths
5 years	30	+1,014 (+0 %)	+9,044 (+34 %)	+2,572 (+29 %)	+308 (+42 %)	+1,651 (+59 %)	+722 (+36 %)	−71 (−2 %)	−114 (−29 %)	−32 (−27 %)
	25	+1,455 (+0 %)	+17,741 (+54 %)	+3,743 (+31 %)	+497 (+50 %)	+2,311 (+58 %)	+1,040 (+36 %)	−63 (−2 %)	−116 (−32 %)	−32 (−27 %)
	20	+1,807 (+0 %)	+22,293 (+59 %)	+4,747 (+33 %)	+581 (+51 %)	+3,079 (+60 %)	+1,282 (+37 %)	−153 (−3 %)	−117 (−33 %)	−32 (−27 %)
3 years	30	+1,352 (+0 %)	+11,375 (+30 %)	+2,932 (+26 %)	+442 (+40 %)	2,247 (+55 %)	+652 (+25 %)	−384 (−12 %)	−67 (−20 %)	−19 (−16 %)
	25	+2,136 (+0 %)	+24,759 (+52 %)	+4,332 (+28 %)	+754 (+49 %)	+3,185 (+54 %)	+950 (+25 %)	−530 (−12 %)	−66 (−24 %)	−18 (−18 %)
	20	+2,909 (+0 %)	+32,648 (+58 %)	+5,698 (+30 %)	+931 (+51 %)	+4,403 (+56 %)	+1,184 (+25 %)	−792 (−17 %)	−65 (−27 %)	−17 (−19 %)
2 years	30	+1,980 (+0 %)	+12,642 (+23 %)	+3,220 (+24 %)	+609 (+38 %)	+2,756 (+49 %)	+437 (+14 %)	−566 (−18 %)	−41 (−15 %)	−12 (−13 %)
	25	+3,204 (+0 %)	+31,715 (+47 %)	+4,780 (+25 %)	+1,072 (+47 %)	+3,891 (+49 %)	+631 (+14 %)	−798 (−19 %)	−39 (−19 %)	−11 (−14 %)
	20	+4,023 (+0 %)	+45,199 (+59 %)	+6,316 (+27 %)	+1,373 (+51 %)	+5,367 (+50 %)	+734 (+13 %)	−1,142 (−26 %)	−39 (−18 %)	−12 (−13 %)
1 year	30	+3,719 (+0 %)	+14,271 (+14 %)	+3,477 (+19 %)	+1,093 (+35 %)	+3,113 (+34 %)	−140 (−4 %)	−584 (−22 %)	−18 (−8 %)	−7 (−9 %)
	25	+6,113 (+0 %)	+51,316 (+43 %)	+5,361 (+21 %)	+2,046 (+47 %)	+4,330 (+34 %)	−200 (−4 %)	−811 (−23 %)	−17 (−10 %)	−7 (−9 %)
	20	+8,211 (+0 %)	+76,480 (+55 %)	+7,259 (+22 %)	+2,675 (+50 %)	+6,096 (+35 %)	−400 (−6 %)	−1,108 (−35 %)	−17 (−10 %)	−6 (−9 %)

Numbers are differences between primary cytology and primary HPV screening, shown separately in Supplementary Tables 1 and 2

*CIN* cervical intraepithelial neoplasia

The table shows undiscounted numbers per 100,000 simulated women, with percentage changes between brackets

^a^As compared to primary cytology, the number of primary screens is slightly higher for HPV screening (i.e., less than 1 %) because it detects more (progressive) CIN lesions, resulting in fewer women being diagnosed with cervical cancer. Whereas women who have been diagnosed with a CIN lesion are assumed to be referred back to routine screening, those with cervical cancer are not

#### Frequent screening from age 30

**Fig. 1:**
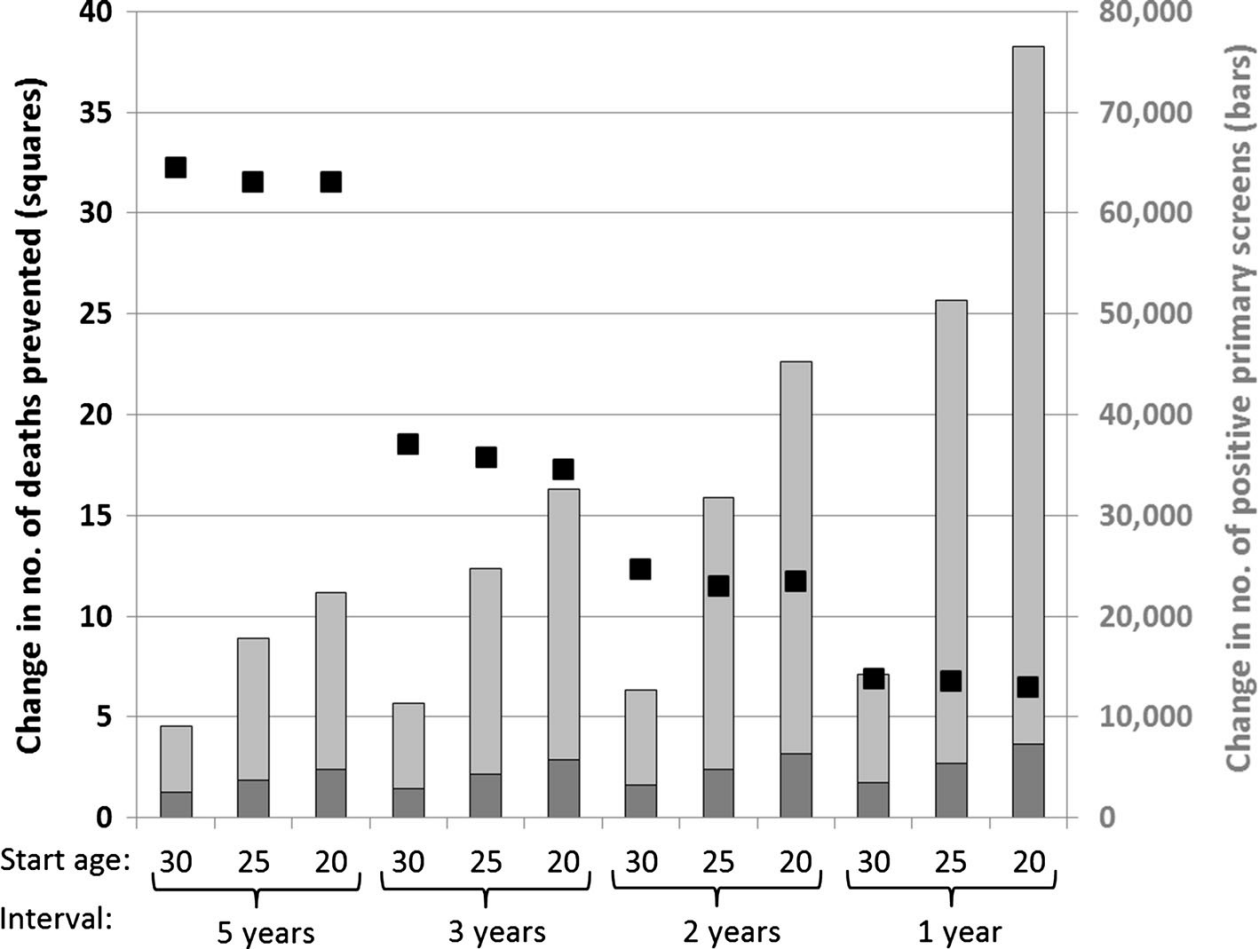
Simulated increase in lifetime number of deaths from cervical cancer prevented (*left axis*) and positive primary screens (*right axis*) when primary cytology is replaced with primary HPV screening. The increase in positive primary tests is split up in referrals to colposcopy (*dark gray*) and non-referrals to colposcopy (*light gray*). Undiscounted results for different start ages and intervals of screening are given per 100,000 women

For 5-yearly screening starting at age 30, replacing primary cytology with primary HPV screening reduced the number of cervical cancer deaths by 32 per 100,000 simulated women, which was a reduction of 27 % (Fig. 1; Table 3). This reduction was achieved at the expense of 9,044 more positive primary screens per 100,000 women (+34 %), resulting in 2,572 more referrals to colposcopy (+29 %). With annual screening in the same age range, switching to primary HPV screening would prevent only 7 extra deaths per 100,000 women (−9 %), while positive primary screens would increase by 14,271 (+14 %) and referrals to colposcopy by 3,477 (+19 %). The (discounted) NNF was 769 in the first scenario versus 11,880 in the latter, more intensive one (Table 4).

**Table 4:** Number of additional positive primary screens per additionally prevented cervical cancer death (NNF) when primary cytology is replaced with primary HPV screening, for the base case and eight sensitivity analyses

Screening interval	Start age	Base case	Background risk of cervical cancer mortality ↑	HPV prevalence in CIN grade I or less^a^ ↑	Sensitivity of cytology ↓	Specificity of cytology ↓	Sensitivity of HPV test ↑	Sensitivity of HPV test ↓	Cytology triaged as in Dutch program	No discounting	5 % discounting
5 years	30	769	399	887	503	NA^b^	805	761	657	280	1,256
	25	1,589	811	1,788	993	502	1,628	1,638	1,360	562	2,692
	20	2,065	1,057	2,352	1,309	747	2,117	2,108	1,772	706	3,603
3 years	30	1,889	969	2,202	1,190	NA^b^	2,047	1,690	1,532	613	3,223
	25	4,443	2,289	4,997	2,712	1,009	4,725	4,213	3,645	1,385	7,927
	20	6,444	3,275	7,324	3,827	1,980	6,907	6,088	5,282	1,886	12,056
2 years	30	3,865	2,006	4,531	2,545	NA^b^	4,360	3,197	2,983	1,027	7,182
	25	10,526	5,341	11,779	6,443	1,346	11,521	9,381	8,140	2,760	20,582
	20	15,405	7,764	17,331	9,506	4,229	16,723	13,750	11,941	3,850	32,252
1 year	30	11,880	6,220	13,731	9,024	NA^b^	14,088	8,562	NA^c^	2,073	27,408
	25	36,576	18,503	39,954	28,544	NA^b^	40,654	30,861	NA^c^	7,570	86,763
	20	60,133	29,372	65,790	45,416	NA^b^	66,783	50,634	NA^c^	11,788	156,829

*HPV* human papillomavirus; *CIN* cervical intraepithelial neoplasia; *NA* not applicable

Numbers were discounted with an annual rate of 3 %, unless stated otherwise

^a^The number of women with a false-positive result or CIN grade I was doubled to account for a higher HPV prevalence among these women

^b^The number of positive primary screens decreased with switching to HPV screening

^c^The current Dutch screening program involves triage testing at 18 months after the primary test, which, for annual screening, interferes with the next screening round

#### Frequent screening from age 20

With annual screening starting at the age of 20 instead of 30, switching from primary cytology to primary HPV screening resulted in similar benefits [i.e., six additional deaths prevented per 100,000 women (−9 %)]. However, the number of women with a positive screen test increased by 76,480 instead of by 14,271 per 100,000 women. The NNF equaled 60,133, which was more than 5 times the NNF of switching in case of annual screening from age 30 and more than 78 times the NNF of switching in case of 5-yearly screening from age 30.

#### Changes in QALYs

Table 5 shows the QALYs gained (or lost) by switching from primary cytology to primary HPV screening for the diverse screening behaviors. Under base case assumptions, a substantial number of QALYs were gained for women who were screened every 5 years from age 30. For more intensively screened women, the benefit of switching to HPV screening was uncertain. For women screened annually or biennially from any age, or triennially from age 20 or 25, replacing primary cytology with primary HPV testing even resulted in a net health loss.

**Table 5:** Simulated change in number of QALYs gained and percentage change when primary cytology is replaced with primary HPV screening, for the base case and eight sensitivity analyses

Screening interval	Start age	Base case	Background risk of cervical cancer mortality ↑	HPV prevalence in CIN grade I or less^a^ ↑	Sensitivity of cytology ↓	Specificity of cytology ↓	Sensitivity of HPV test ↑	Sensitivity of HPV test ↓	Cytology triaged as in Dutch program	No discounting	5 % discounting
5 years	30	+116 (+4 %)	+319 (+5 %)	+101 (+3 %)	+265 (+9 %)	+115 (+4 %)	+130 (+4 %)	+80 (+2 %)	+175 (+20 %)	+667 (+5 %)	+27 (+2 %)
	25	+57 (+2 %)	+258 (+4 %)	+34 (+1 %)	+203 (+7 %)	+58 (+2 %)	+63 (+2 %)	+37 (+1 %)	+124 (+15 %)	+591 (+4 %)	−26 (−2 %)
	20	+16 (+1 %)	+214 (+3 %)	−19 (−1 %)	+153 (+5 %)	+16 (+1 %)	+17 (+1 %)	+3 (+0 %)	+98 (+13 %)	+559 (+4 %)	−70 (−6 %)
3 years	30	+4 (+0 %)	+105 (+2 %)	−16 (−0 %)	+83 (+3 %)	+4 (+0 %)	+4 (+0 %)	−2 (−0 %)	+72 (+8 %)	+254 (+2 %)	−27 (−2 %)
	25	−67 (−2 %)	+24 (+0 %)	−99 (−3 %)	−6 (−0 %)	−66 (−2 %)	−75 (−2 %)	−59 (−2 %)	+16 (+2 %)	+157 (+1 %)	−89 (−7 %)
	20	−125 (−4 %)	−37 (−1 %)	−175 (−6 %)	−71 (−2 %)	−124 (−4 %)	−138 (−4 %)	−111 (−4 %)	−15 (−2 %)	+88 (+1 %)	−147 (−13 %)
2 years	30	−52 (−2 %)	+2 (+0 %)	−76 (−2 %)	−19 (−1 %)	−52 (−2 %)	−57 (−2 %)	−49 (−1 %)	+34 (+4 %)	+56 (+0 %)	−55 (−4 %)
	25	−133 (−4 %)	−86 (−1 %)	−172 (−5 %)	−114 (−4 %)	−132 (−4 %)	−144 (−4 %)	−117 (−4 %)	−20 (−2 %)	−55 (−0 %)	−125 (−10 %)
	20	−196 (−6 %)	−148 (−2 %)	−257 (−9 %)	−189 (−6 %)	−195 (−6 %)	−210 (−7 %)	−174 (−6 %)	−41 (−7 %)	−112 (−1 %)	−191 (−19 %)
1 year	30	−127 (−4 %)	−106 (−2 %)	−155 (−5 %)	−128 (−4 %)	−125 (−4 %)	−135 (−4 %)	−115 (−4 %)	NA^b^ (−)	−154 (−1 %)	−99 (−8 %)
	25	−238 (−8 %)	−218 (−3 %)	−283 (−10 %)	−254 (−8 %)	−237 (−8 %)	−253 (−8 %)	−214 (−7 %)	NA^b^ (−)	−290 (−2 %)	−197 (−19 %)
	20	−334 (−12 %)	−315 (−5 %)	−407 (−15 %)	−360 (−13 %)	−332 (−12 %)	−354 (−13 %)	−303 (−11 %)	NA^b^ (−)	−390 (−3 %)	−293 (−36 %)

*HPV* human papillomavirus, *CIN* cervical intraepithelial neoplasia, *NA* not applicable

The table shows numbers per 100,000 simulated women, with percentage changes between brackets. Numbers were discounted with an annual rate of 3 %, unless stated otherwise

^a^The number of women with a false-positive result or CIN grade I was doubled to account for a higher HPV prevalence among these women

^b^The current Dutch screening program involves triage testing at 18 months after the primary test, which, for annual screening, interferes with the next screening round

### Sensitivity analyses

In all sensitivity analyses, primary HPV screening prevented more cervical cancer deaths than did primary cytology. In most scenarios, this occurred at the expense of more positive screens, and the NNF increased quite rapidly with the intensity of the screening scenario (Table 4). Only when the specificity of cytology was assumed to be lower (95.2 % instead of 97.6 %), for some levels of over-screening, the number of positive screens decreased with the shift to primary HPV testing. The discount rate appeared to have the largest impact on the NNF.

In the sensitivity analyses, switching to primary HPV testing resulted in fewer QALYs gained in the case of more intensive screening. Overall, for a given level of over-screening, whether QALYs were gained or lost did not vary substantially among the sensitivity analyses. Generally, switching was favorable for women screened every 5 years and unfavorable for those screened annually or biennially. However, when the background risk of cervical cancer mortality was increased, when cytology was triaged as is currently recommended in the Dutch screening program or when health effects were not discounted, switching to HPV screening also resulted in QALYs gained for women screened biennially from age 30. For women screened every 5 years from age 20, QALYs were lost when the HPV prevalence was increased and when results were discounted at an annual rate of 5 %.

## Discussion

Even in countries with carefully constructed screening guidelines, women may be over-screened. As for over-screened women the risk of cervical cancer is already strongly reduced with primary cytology, the gains of switching to primary HPV screening are expected to be relatively small. Indeed, our analysis predicted that while switching would prevent 32 deaths per 100,000 women who are screened every 5 years, only 6–7 deaths would be averted in those screened annually. In the latter group, the increase in positive tests and subsequent follow-up procedures even resulted in a net loss in health.

Because the same conclusion was reached in all of the sensitivity analyses, it is likely generalizable to other developed countries. The lower the ratio of HPV prevalence to cervical cancer mortality risk, the less harmful the HPV testing will be for over-screened women. The sensitivity analysis in which we doubled the lifetime risk of dying from cervical cancer showed that it would still be harmful if this ratio would be twice as low as in the Netherlands though. In countries with an even lower HPV prevalence to cervical cancer mortality risk ratio, switching to HPV testing might be beneficial for over-screened women. In the USA, however, both HPV prevalence and cervical cancer mortality are comparable to the Netherlands [37, 38]. In most European countries, cervical cancer mortality is higher [39], but HPV prevalence is also (up to) twice as high [38].

Obviously, the goal of a cancer screening program is to decrease the disease’s incidence and mortality rate. Because in every simulated scenario switching from primary cytology to primary HPV screening reduced the number of cervical cancer cases and deaths, one could argue that primary HPV screening should always be preferred. This would indeed be true if being in triage, being referred for colposcopy, and being treated for CIN would not be associated with losses in quality of life. However, the health-related burden of these events is a drawback of screening that should not be overlooked [40, 41].

A number of randomized controlled trials (RCTs) have compared primary cytology screening to either HPV screening alone or HPV screening combined with cytology [42–45]. In these RCTs, HPV screening resulted in a higher detection rate of CIN lesions and an improved protection against cervical cancer [46]. CEAs based on these findings showed that primary HPV screening with an interval of at least 3 years is cost-effective for women above age 30 [9, 47]. We showed that the effectiveness is questionable if this cannot be guaranteed. In this regard, data from a US population-based registry showed that recommending 3-yearly cytology screening resulted in a median time between two consecutive smears of 1.87 years in 2011 [48]. There is no reason to assume that guidelines regarding primary HPV screening would be followed more closely. In fact, a study from 2010 found a lower adherence to guidelines after a negative co-test as compared to after a negative cytological test [19]. Although co-testing is intended for women who want to extend their screening interval from 3 to 5 years, many clinicians provide it on an annual basis [19].

Switching to HPV screening could be considered more effective for women with that level of over-screening for which HPV screening was associated with a net health benefit, but this would not necessarily be more cost-effective. However, the decision to include primary HPV screening in national screening guidelines should take into account its population-level cost-effectiveness. If only a relatively small number of women are over-screened, then switching to HPV screening may well be (very) cost-effective on a population level. In the Netherlands, given the small number of smears taken outside the screening program [49], it is expected to be cost-effective.

### Strengths and limitations

Even though earlier research showed that primary HPV screening is more cost-effective than primary cytology for women who adhere to screening guidelines [10, 50], this is the first study to quantify its harms and benefits for over-screened women. As over-screening practices are likely to remain, these results are relevant to any country considering recommending primary HPV screening, either alone or as a co-test.

Our study also has some limitations. First of all, our model is based on Dutch data. Although it might have been better to adjust the model for every single country, we did vary those country-specific parameters that would influence the conclusion. For example, we increased the HPV prevalence level to estimate effects for high HPV prevalence countries such as Denmark [51]. We did not modify the prevalence age distribution as the peak between the ages of 20 and 30 has also been observed in other European countries and in the USA [51, 52].

Although we varied test characteristics to explore the effect of switching to HPV screening for different settings, the ranges considered are not representative for low- and middle-income countries, where sustaining cytology programs of sufficient quality is often difficult [53, 54]. As the test characteristics are only one of many factors that may be different in those countries, separate analyses are needed for these situations.

Meta-analyses have shown that removal of CIN lesions carries an increased risk of having preterm births [55, 56]. We did not include this potential harm because estimates of the impact on a woman’s quality of life are unavailable. If we would have accounted for this in our analyses, in over-screened women even more QALYs would have been lost by switching to primary HPV screening.

Although there are numerous possible triage strategies for cytology and HPV testing, in the base case analysis we only considered two that were found to be cost-effective in a previous analysis [9]. In a sensitivity analysis, we did explore the impact of switching from the less efficient cytology screening strategy that is currently recommended in the Netherlands to the cost-effective HPV screening strategy that will be implemented in 2017. When these less efficient cytology practices were assumed, switching to HPV testing was obviously more beneficial. Nevertheless, it still resulted in a net health loss for women screened biennially from age 20 to 25 or triennially from age 20 (effects for annually screened women were not evaluated for this triage strategy). If future triage practices would be much more efficient than current ones, then switching to HPV testing might be considered beneficial for over-screened women, but this would be due to more efficient triage procedures rather than to an improved performance of the primary test.

Lastly, we did not consider a co-testing strategy, which is already recommended in the USA for women aged 30–65 years [12, 17, 57]. Co-testing results in more screen positives than does primary HPV screening because HPV negative smears can still be cytology positive. From results of an RCT performed in the Netherlands, where women aged 30–60 years are screened every 5 years, we calculated that the number of screen positives would be 33 % higher with co-testing than with primary HPV screening [28]. As a consequence, the number of screen-detected CIN grade III lesions or cancer would be 7 % higher. In an RCT performed in the UK, in which women aged 20–64 years were screened with an interval of 2–4 years, the number of screen positives would have been 46 % higher with co-testing as compared to primary HPV screening, while the number of screen-detected clinically relevant lesions (at least CIN grade III) would have been only 3 % higher [58]. For intensively screened women, co-testing can potentially prevent slightly more cervical cancer cases than primary HPV screening, but the utility loss associated with the additional positive screens probably outweighs these minor gains. Therefore, co-testing is expected to be even more harmful than primary HPV screening alone for over-screened women.

## Conclusion

We determined the pros and cons of replacing primary cytology with primary HPV screening for women who are over-screened, i.e., from a younger age and with a shorter screening interval than recommended. Although in all scenarios more deaths would be averted by screening primarily with the HPV test, the negative effects outweighed the benefits. We may conclude that irrespective of costs, it is disputable to recommend primary HPV screening, either alone or as a co-test, as long as a substantial part of the population is still over-screened. A well-organized and structurally monitored screening program, in which primary tests taken outside the program are not reimbursed by the government, could help minimizing the number of tests taken outside the program, thereby limiting the level of over-screening [21, 59]. One may consider to first further develop strategies to reduce over-screening or at least give it high priority when issuing guidelines including primary HPV screening.

## Electronic supplementary material

Supplementary material 1 (DOCX 293 kb)

Supplementary material 2 (DOCX 39 kb)

Supplementary material 3 (DOCX 41 kb)
