# Supplementary material for: The potential harms of primary human papillomavirus screening in over-screened women: a microsimulation study
Source: Cancer Causes Control. 2016 Mar 12;27:569–81. doi: 10.1007/s10552-016-0732-7 (PMC4796367; doi:10.1007/s10552-016-0732-7)
Supplement: Supplementary file 2 — Supplementary material 2 (DOCX 39 kb) [file 10552_2016_732_MOESM2_ESM.docx]

**Supplementary Table 1. Effects of primary cytology for 12 different screening scenarios; undiscounted numbers per 100,000 simulated women.**

| **Screening**  **interval** | **Start**  **age** | **# Primary**  **screens** | **# Positive primary screens** | **# Referrals** | **# False-positive referrals (no CIN detected)** | **# CIN grade I** | **# CIN grade II** | **# CIN grade III** | **# Cervical cancer cases** | **# Cervical cancer deaths** |
| --- | --- | --- | --- | --- | --- | --- | --- | --- | --- | --- |
| 5 years | 30 | 739,525 | 26,923 | 8,868 | 742 | 2,819 | 2,015 | 3,174 | 398 | 121 |
|  | 25 | 840,937 | 32,721 | 12,139 | 988 | 3,980 | 2,893 | 4,177 | 358 | 116 |
|  | 20 | 941,786 | 37,492 | 14,305 | 1,148 | 5,144 | 3,451 | 4,465 | 352 | 115 |
| 3 years | 30 | 1,118,531 | 38,434 | 11,168 | 1,108 | 4,106 | 2,609 | 3,268 | 332 | 114 |
|  | 25 | 1,315,382 | 47,706 | 15,619 | 1,540 | 5,868 | 3,778 | 4,377 | 269 | 100 |
|  | 20 | 1,511,580 | 56,297 | 19,192 | 1,838 | 7,934 | 4,722 | 4,658 | 244 | 91 |
| 2 years | 30 | 1,674,942 | 54,320 | 13,581 | 1,609 | 5,634 | 3,110 | 3,172 | 272 | 96 |
|  | 25 | 1,971,111 | 66,941 | 19,192 | 2,259 | 8,003 | 4,489 | 4,238 | 209 | 82 |
|  | 20 | 2,183,094 | 76,854 | 23,484 | 2,703 | 10,818 | 5,618 | 4,321 | 215 | 90 |
| 1 year | 30 | 3,346,636 | 99,553 | 18,556 | 3,116 | 9,033 | 3,658 | 2,708 | 222 | 79 |
|  | 25 | 3,858,503 | 118,972 | 25,785 | 4,380 | 12,625 | 5,236 | 3,530 | 176 | 75 |
|  | 20 | 4,368,263 | 138,478 | 32,507 | 5,377 | 17,425 | 6,540 | 3,159 | 166 | 75 |

CIN = cervical intraepithelial neoplasia.
