# Supplementary material for: The potential harms of primary human papillomavirus screening in over-screened women: a microsimulation study
Source: Cancer Causes Control. 2016 Mar 12;27:569–81. doi: 10.1007/s10552-016-0732-7 (PMC4796367; doi:10.1007/s10552-016-0732-7)
Supplement: Supplementary file 3 — Supplementary material 3 (DOCX 41 kb) [file 10552_2016_732_MOESM3_ESM.docx]

**Supplementary Table 2. Effects of primary HPV screening for 12 different screening scenarios; undiscounted numbers per 100,000 simulated women.**

| **Screening**  **interval** | **Start age** | **# Primary**  **screens** | **# Positive primary screens** | **# Referrals** | **# False-positive referrals (no CIN detected)** | **# CIN grade I** | **# CIN grade II** | **# CIN grade III** | **# Cervical cancer cases** | **# Cervical cancer deaths** |
| --- | --- | --- | --- | --- | --- | --- | --- | --- | --- | --- |
| 5 years | 30 | 740,539 | 35,968 | 11,440 | 1,050 | 4,470 | 2,737 | 3,103 | 283 | 89 |
|  | 25 | 842,392 | 50,462 | 15,881 | 1,485 | 6,290 | 3,933 | 4,113 | 242 | 84 |
|  | 20 | 943,593 | 59,784 | 19,052 | 1,730 | 8,223 | 4,733 | 4,312 | 234 | 84 |
| 3 years | 30 | 1,119,883 | 49,809 | 14,100 | 1,550 | 6,353 | 3,261 | 2,883 | 265 | 95 |
|  | 25 | 1,317,518 | 72,465 | 19,951 | 2,294 | 9,053 | 4,729 | 3,847 | 204 | 82 |
|  | 20 | 1,514,489 | 88,945 | 24,890 | 2,769 | 12,327 | 5,907 | 3,866 | 179 | 74 |
| 2 years | 30 | 1,676,921 | 66,962 | 16,802 | 2,218 | 8,391 | 3,546 | 2,605 | 231 | 83 |
|  | 25 | 1,974,315 | 98,656 | 23,801 | 3,330 | 11,894 | 5,121 | 3,440 | 170 | 71 |
|  | 20 | 2,187,117 | 122,053 | 29,800 | 4,075 | 16,185 | 6,351 | 3,179 | 176 | 79 |
| 1 year | 30 | 3,350,355 | 113,824 | 22,033 | 4,209 | 12,146 | 3,519 | 2,124 | 205 | 72 |
|  | 25 | 3,864,616 | 170,288 | 31,145 | 6,426 | 16,955 | 5,036 | 2,719 | 158 | 68 |
|  | 20 | 4,376,474 | 214,958 | 39,766 | 8,052 | 23,521 | 6,140 | 2,051 | 149 | 68 |

CIN = cervical intraepithelial neoplasia.
